# Outcomes of Percutaneous Versus Open Lumbopelvic Fixation of Spinopelvic Dissociation

**DOI:** 10.1155/aort/9946662

**Published:** 2025-07-15

**Authors:** Sean Taylor, Saurabh Rawall, Asa Peterson, Gerald McGwin, Sakthivel Rajaram

**Affiliations:** ^1^Department of Orthopaedic Surgery, University of Alabama at Birmingham, 1313 13^th^ Street South, Birmingham 35205-5327, Alabama, USA; ^2^Department of Epidemiology, University of Alabama at Birmingham, 1720 University Blvd, Birmingham 35233-1816, Alabama, USA

**Keywords:** lumbopelvic fixation, percutaneous, spinopelvic dissociation, transverse sacral fracture, u-type sacral fracture

## Abstract

**Introduction:** Spinopelvic dissociation is a devastating injury that remains difficult to manage due to its complexity and low incidence. Lumbopelvic fixation is a treatment option traditionally performed with an open approach. However, open fixation is associated with substantial blood loss and infection risk in critical polytrauma patients. Technological advancements have enabled this procedure to be performed percutaneously. Thus, we evaluate outcomes between patients receiving open lumbopelvic fixation and those receiving percutaneous lumbopelvic fixation.

**Methods:** A retrospective review was conducted of patients undergoing either open or percutaneous lumbopelvic fixation for spinopelvic dissociation from 2012 to 2024. The AOSpine classification system was used to classify all fractures. Patient demographic, clinical, and operative outcomes were analyzed.

**Results:** 48 patients with spinopelvic dissociation were included in the final analysis, with 21 receiving open lumbopelvic fixation and 27 receiving percutaneous lumbopelvic fixation. Preoperative characteristics and demographics were similar between the two groups. The percutaneous group demonstrated significantly reduced blood loss (82 vs. 679 mL; *p* < 0.01), shorter operative time (168 vs. 284 min; *p* < 0.01), fewer surgical site infections (0 vs. 4; *p*=0.03), and reduced OR cost ($35,097 vs. $23,743; *p*=0.01) but had a higher rate of anterior pelvic ring injuries (63% vs. 19%; *p*=0.003). There was no significant difference in length of stay (*p*=0.63) or length of follow-up (*p*=0.64).

**Conclusion:** Our findings suggest that percutaneous lumbopelvic fixation offers an attractive less invasive and shorter procedure to treat spinopelvic dissociation without added morbidity.

## 1. Introduction

Spinopelvic dissociation is a result of the discontinuation of the spine and posterior pelvic ring. It is a rare and complex injury that usually results from high-energy mechanisms and has been termed the “suicide jumper's fracture [1].” Sacral fractures resulting in spinopelvic dissociation are usually multiplanar, differentiating these fractures from fracture dislocations at the lumbosacral junction or bilateral sacroiliac fractures [2]. Classifications combining vertical and horizontal fracture patterns, such as H-, U-, T-, and Y-type, have been proposed based on morphological characteristics [3]. Transverse sacral fractures account for 3%–5% of all pelvic ring traumas, and an even smaller percentage of those (2.9%) result in spinopelvic dissociation [4, 5].

Despite its rarity, spinopelvic dissociation can cause significant morbidity. Neurological injury is associated with up to 68% of spinopelvic dissociation fractures [6]. Moreover, these injuries are usually encountered in polytrauma patients. Associated spine fractures, lower extremity fractures, contiguous pelvic fractures, and visceral injuries are seen in 29%–88% of spinopelvic dissociation patients [7]. Given the associated injuries and complexity of the sacral injury pattern, spinopelvic dissociation is commonly a missed or late diagnosis [8]. Thus, a high index of suspicion is key for appropriately identifying and optimizing treatment plans for these patients.

Management of spinopelvic dissociation was traditionally nonoperative due to limited stabilization capability [9, 10]. Nonoperative management is indicated in select circumstances, including nondisplaced high transverse fractures of less than 10 mm, sacral fractures below the sacroiliac joint, and patients who will be nonambulatory for 2–3 months either due to comorbidities, preexisting conditions, or concomitant injuries [6, 11–13]. Due to technical advancements and improvements in diagnosis, surgical intervention of spinopelvic dissociation has superseded nonoperative management. Operative management is associated with improved long-term outcomes regarding quality of life, such as mood and pain control [10].

Surgical procedures for these crucial injuries aim to restore the continuity of the axial spine to the posterior pelvic ring. Surgery facilitates earlier mobilization, which is crucially important for patients with spinopelvic dissociation and commonly associated injuries. Surgical options include iliosacral or transsacral screws, lumbopelvic fixation, or triangular osteosynthesis [7, 10, 11, 14–16]. Each technique offers particular advantages, but patient-specific factors and surgeon comfort are most important. Open reduction with internal lumbopelvic fixation has been used traditionally to manage spinopelvic dissociation; however, it is associated with a high rate of postoperative infections, ranging from 14% to 16% [10, 14, 17–19]. Percutaneous lumbopelvic fixation is another approach that offers a lower infection rate comparatively [14, 20, 21]. Moreover, percutaneous lumbopelvic fixation allows for earlier mobilization [15]. The importance of early mobilization in these usually polytraumatized patients cannot be overstated. Despite these advantages, it may be challenging for surgeons to develop expertise using minimally invasive techniques, given the complexity and rarity of spinopelvic dissociation presentations. A comparative analysis of open and minimally invasive procedures can help evaluate the benefits and feasibility of the percutaneous approach. Thus, we intend to assess the outcomes of spinopelvic dissociation treated with open reduction or percutaneous lumbopelvic fixation.

## 2. Methods

After institutional review board approval was obtained, a retrospective review was conducted on operatively managed spinopelvic dissociations at a level I trauma center from January 2012 to November 2024. Patients over 18 years of age who underwent spinopelvic fixation were included, and patients with preoperative lower lumbar, sacral, or pelvic hardware, and transsacral screws were excluded. Patient characteristics and demographic information were collected, including age, gender, BMI, mechanism of injury, associated injuries, neurological injury, and tobacco use. The fractures were classified according to the Arbeitsgemeinschaft für Osteosynthesefragen (AO) sacrum classification. M3 (anterior pelvic ring injury) and M4 (sacroiliac joint injury) modifiers were considered for further pelvic x-ray subclassification.

Measured clinical outcomes included surgical site infection, transfusions within 72 h postoperatively, estimated blood loss, and operative time. Postoperative parameters including hardware removal and spinopelvic measurements (lumbar lordosis and pelvic incidence) were collected. Hardware removal was recorded and stratified by underlying cause, including elective removal, symptomatic hardware prominence, screw loosening, and postoperative complications such as dural tear and infection. Both the fracture classification and spinopelvic measurements were decided by interobserver agreement between two individuals. All patients were allowed to bear weight as tolerated unless accompanying lower extremity fractures prohibited this.

### 2.1. Surgical Technique

All procedures were performed by spine fellowship-trained orthopedic surgeons during the inpatient stay. The decision to use open versus percutaneous fixation was made at the discretion of the attending surgeon based on factors such as the presence of associated injuries, the location of potential incision sites, body habitus, and the surgeon's experience. The percutaneous procedures involved indirect reduction and lumbar four or five pedicle screws with S2-alar-iliac (S2AI) fixation, as described by Wang et al. [22]. Neurological dysfunction was treated with either laminectomies or fracture reduction with indirect decompression.

For the percutaneous procedures, patients were positioned prone with bumps under the thighs to create an extension force on the legs. This thigh extension was the main reduction maneuver. Bilateral L5 pedicles were cannulated with Jamshidi needles under fluoroscopic guidance. Thereafter, screws were passed over guide wires. A single midline 1-inch skin incision was used for both S2AI screws, and fascial cuts were made about 1 inch on either side of the midline. Jamshidi needles were also used for S2AI screw fixation. The initial needle placement was ventromedial to the posterior superior iliac spine (PSIS). Using a teardrop view, the Jamshidi needle was guided down between the inner and outer tables of the pelvis. AP/inlet/outlet radiographs were then obtained to ensure no violation of the sciatic notch. Thereafter, S2AI screws were passed over the guide wires. This was followed by rods and set screw placement. In the author's experience, placing and tightening unilateral instrumentation before starting with the contralateral instrumentation makes the procedure technically less demanding.

### 2.2. Statistical Analysis

Demographic, clinical, and outcome variables were compared using Fisher's exact test for categorical data and analysis of variance (ANOVA) for continuous data. Two-sided *p* values ≤ 0.05 were considered statistically significant. All statistical analyses were performed using SAS software.

## 3. Results

48 patients with spinopelvic dissociation were included in the final analysis, with 21 receiving open reduction and lumbopelvic fixation and 27 receiving percutaneous lumbopelvic fixation. Age, gender, BMI, and smoking status were similar between the two groups, as depicted in Table 1. The open group had an average of 16.9 days in the hospital from admission to discharge, while the percutaneous group averaged 18.2 days (*p*=0.63). The average outpatient follow-up was 11.8 months for the open group and 10.4 months for the percutaneous group (*p*=0.64).

AO classifications were assigned to injury patterns, as shown in Table 2 [23]. Fractures were classified as nondisplaced U-type (C0), unilateral B-type fractures (C1), bilateral B-type fractures without a transverse component (C2), and displaced U-type fractures (C3). Anterior pelvic ring injuries (M3) and sacroiliac joint injuries (M4) were used as modifiers in the fracture classifications. In the open group, 6 were classified as C0, 1 as C1, and 14 as C3. In the C3 group, 3 had the M3 qualifier, 1 had the M4 qualifier, and 1 had both qualifiers. In the percutaneous group, 7 were classified as C0, 2 as C1, and 18 as C3. In the C1 fractures, one had an M3 modifier, and another had both modifiers. In the C3 fractures, 10 had an M3 modifier, and 3 had both modifiers. These classifications are depicted in Tables 2 and 3.

Associated injuries, classified as traumatic brain injury, closed head injury, extremity fracture, anterior pelvic ring fracture, acetabulum fracture, thoracic injury, blunt abdominal injury, and associated spine injury, are shown in Table 4. Extremity fractures requiring surgery were observed in 65% of the 48 patients. A larger percentage of the open cohort had traumatic brain injury, thoracic injury, and associated spine injury compared to the percutaneous group. All associated injuries were similar between groups, except for anterior pelvic ring injury, which was significantly more common in the percutaneous cohort (*p*=0.003)

Blood loss was significantly higher (*p* < 0.01) in the open group (679 mL) compared to the percutaneous group (82 mL). Operative time was significantly longer (*p* < 0.01) for the open group (284 min) compared to the percutaneous group (168 min). Transfusions within a 72-h postoperative window were seen in 6 patients (29%) treated with open fixation and 8 (30%) in the percutaneous group (*p*=0.94). Postoperative spinopelvic radiographic parameters (lumbar lordosis and pelvic incidence) were collected. The average lordosis was 55.3° in the open group and 51.2° in the percutaneous group (*p*=0.20). The average pelvic incidence was 68.9° in the open group and 65.1° in the percutaneous group (*p*=0.15). 5 patients in the open group had hardware removed compared to 11 in the percutaneous group (*p*=0.35). Of the 5 hardware removals in the open cohort, one was elective, one followed a dural tear, two were due to infection, and one was related to screw loosening. Of the 11 hardware removals in the percutaneous cohort, 5 were elective, 4 were for symptomatic hardware prominence, and 2 were due to postoperative pain.

All 4 surgical site infections were observed in the open group (*p*=0.03). The average OR cost was significantly higher (*p*=0.01) for open procedures ($35,097) compared to the percutaneous group ($23,743). These operative and postoperative outcomes are shown in Table 5.

## 4. Discussion

**Figure 1:**
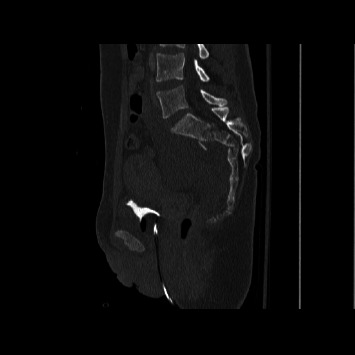
Sagittal CT showing an AOSpine type C3 sacral fracture.

**Figure 2:**
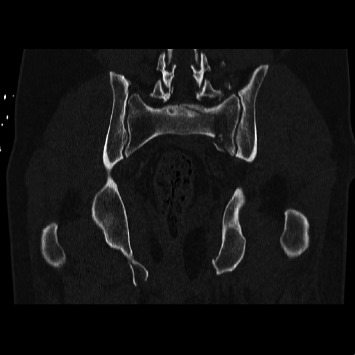
Coronal preoperative CT showing multiplanar sacral fracture resulting in spinopelvic discontinuity.

Spinopelvic dissociation is a devastating injury that remains difficult to manage due to its low incidence and complexity. These multiplanar sacral fractures are often caused by significant axial loading leading to dissociation, as depicted in Figures 1 and 2. Our understanding of sacral fractures continues to evolve with the development and refinement of classification systems. Denis et al. proposed a classification delineating three zones in the coronal plane of the sacrum and the associated risk of neurological injury [24]. Roy-Camille et al. classified injury patterns based on sagittal imaging and the relationship between the cranial and caudal portions of the sacrum [1]. Lehman et al. devised the lumbosacral injury classification system (LSICS) to aid in classifying and determining optimum operative techniques based on injury classifications [25]. AOSpine created the sacral injury classification system, which integrates fracture morphology, ligamentous complex integrity, neurological status, and clinical modifiers [23]. All of the patients sustained type C AOSpine injuries. 32 out of 48 patients had C3 injuries, which denote displaced u-type fractures.

**Figure 3:**
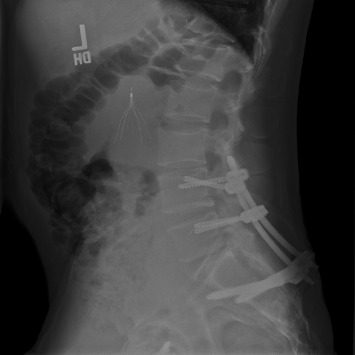
Postoperative X-ray in a patient who underwent open reduction and lumbopelvic fixation.

Given the instability of these injuries, appropriate reduction is crucially important. Indirect reduction maneuvers, as described by Nork et al., were used in percutaneous procedures [4]. Comparable postoperative lumbar lordosis (*p*=0.59) and pelvic incidence (*p*=0.49) were appreciated between the treatment groups. We primarily evaluated spinopelvic parameters rather than fracture reduction, though pelvic incidence adequately assesses sagittal plane reduction and can be a reliable predictor of clinical outcomes [26, 27]. Percutaneous spinal fixation can be safely used following successful indirect reduction [14]. Postoperative imaging with satisfactory spinopelvic alignment is depicted in Figures 3 (open) and 4 (percutaneous).

**Figure 5:**
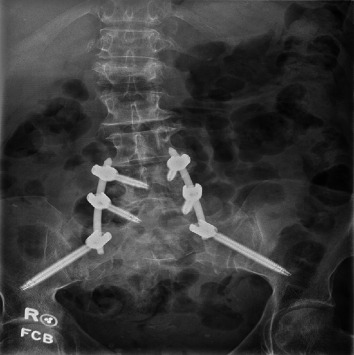
Lumbopelvic fixation extending from L4 to pelvis inserted percutaneously.

Technological advances and increased reporting on spinopelvic dissociation have resulted in diverse operative options for surgeons. Fixation options include either open or percutaneous approaches for transsacral fixation, lumbopelvic fixation, or triangular osteosynthesis. Transsacral fixation horizontally supports the pelvis with compression. This method can augment fixation strength by bilaterally engaging the cortices of the sacroiliac joint. Lumbopelvic fixation was developed to address vertically unstable transforaminal sacral fractures, providing decompression and stable fixation without compression [28]. Spinopelvic fixation offers neutralization and vertical support, maintaining the length of the sacrum and lumbar spine. This neutralization is increased by a wider assembly, and extending fixation to L4 is increasingly recommended, particularly with poor bone quality or accompanied L5 pedicle fracture [12, 13, 29, 30]. This is shown in Figure 5. A combination of horizontal and vertical fixations, or triangular osteosynthesis, offers the most biomechanically stable option [31]. However, stand-alone lumbopelvic fixation offers good clinical outcomes as well [21, 32, 33].

In parallel, recent years have seen the emergence of navigation-guided percutaneous sacroiliac screw fixation as an increasingly adopted alternative, particularly for fractures with predominant horizontal instability. These systems enhance screw placement accuracy and help mitigate neurovascular risk, particularly in patients with complex sacral anatomy or limited fluoroscopic visualization. While not the focus of our present comparison, these modern techniques represent an important advancement in pelvic fixation strategies [20]. One drawback of percutaneous fixation is its reliance on intraoperative fluoroscopy, which increases radiation exposure to both patients and surgical staff. Robotic-assisted fixation has been proposed to mitigate radiation risk by reducing reliance on intraoperative fluoroscopy, though evidence remains inconclusive [34].

In lumbopelvic fixation, the decision to perform lumbar fusion is at the discretion of the surgeon. In instances where fusion is not achieved, hardware removal is recommended to avoid irritation and secondary loss of motion [15, 21]. Ultimately, this second procedure necessitates a shared decision between the surgeon and the patient. At the authors' institution, elective hardware removal is generally offered following confirmation of appropriate bone healing on CT imaging. In our series, all cases of hardware prominence occurred in patients with a BMI below 24, suggesting that variations in body habitus, particularly lower BMI, may influence the likelihood of hardware irritation and the consideration for removal.

Open reduction with lumbopelvic fixation has been associated with a high infection rate [10, 19]. Schidhauer et al. reported an infection rate of 16% with open lumbopelvic fixation [18]. Percutaneous instrumentation has demonstrated low rates of wound complication and decreased blood loss [15, 20, 32]. In this current study, the open group had significantly more blood loss (*p* < 0.001) and more surgical site infections (*p*=0.03). This parallels the previous literature and introduces significant risks for already sick patients. Furthermore, operative time (*p* < 0.001) and OR costs (*p*=0.01) were significantly reduced in the percutaneous group. Macario et al. estimated that each minute of operating room time costs $62 [35]. In addition to cost-effectiveness, the clinical importance of reduced operative time cannot be overstated.

Sacral laminectomy and decompression were performed only in patients with focal compression of sacral nerve roots. Schildhauer et al. found that surgical timing did not correlate with patient outcomes in cauda equina secondary to spinopelvic dissociation [18]. Lindahl et al. similarly found that laminectomy did not improve bladder or bowel dysfunction in patients who underwent decompression [36]. The role of decompression in outcomes remains debated and is beyond the scope of this study. However, a percutaneous approach offers rapid stabilization while preserving the option for subsequent decompression if indicated [4].

There are several limitations to this study. Inherent to retrospective reviews, selection bias plays a role. The surgical approach was determined by surgeon preference, experience, and patient-specific factors such as comorbidities, body habitus, soft tissue condition, and the need for decompression. The rationale behind these decisions is difficult to assess retrospectively, and sacral classification systems, such as the Denis classification, could not be used to stratify neurological risk or correlate with the surgical approach. This may have introduced unmeasured differences in fracture severity or associated injuries between groups. Although we assessed radiographic parameters, our ability to evaluate functional recovery is limited without quality-of-life outcomes such as pain scores, ambulation capacity, and fracture union. Finally, the cost analysis was limited to intraoperative charges and did not account for indirect costs such as rehabilitation, reoperations, or complication management, which may underestimate the true economic impact of each approach. Future prospective studies are needed to assess clinical outcomes, indirect costs, and standardized criteria for surgical selection to better define the comparative value of fixation strategies for spinopelvic dissociation.

As depicted in Table 4, the traumatic injuries sustained were highly diverse. Thus, independently evaluating outcomes of a single procedure and making direct comparisons between the groups are challenging, given the complexity of their multisystem traumatic injuries. Despite being an infrequently encountered injury, larger and better-powered studies can further validate our results. Given the traumatic nature of these injuries, follow-up was inconsistent amongst patients. Despite the open cohort having a lower incidence of long bone and pelvic fractures requiring operative management, they averaged longer follow-ups than the percutaneous group. A more consistent and longer follow-up would provide better insights into the outcomes of lumbopelvic surgical approaches for spinopelvic dissociation.

Our findings suggest that percutaneous lumbopelvic fixation of spinopelvic dissociation offers a quicker procedure to treat these devastating injuries without added morbidity. Percutaneous fixation resulted in a significantly shorter procedure, reduced blood loss, and a lower incidence of surgical site infections. To our knowledge, this is the largest case series directly comparing open and percutaneous lumbopelvic fixation for spinopelvic dissociation. Percutaneous fixation of spinopelvic dissociation can be challenging, given the anatomical complexity and presence of concurrent injuries, and, even with promising results, further research is warranted.

## 5. Conclusion

Our findings suggest that percutaneous lumbopelvic fixation offers an attractive, less invasive, and shorter procedure to treat spinopelvic dissociation without added morbidity.

## Figures and Tables

**Figure 4:**
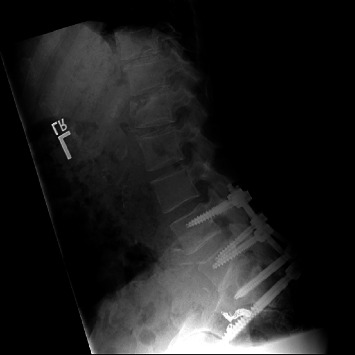
Postoperative X-ray in a patient who underwent percutaneous lumbopelvic fixation.

**Table 1:** Preoperative patient characteristics.

	Open (*n* = 21)	Percutaneous (*n* = 27)	*p* value
Age	49.2	42.6	0.25

Gender	Females: 10 Males: 11	Females: 10 Males: 17	0.56

BMI	25.96	25.10	0.59

Smoking	12 (57%)	12 (44%)	0.56

Cauda equina	3	4	1.0

**Table 2:** Classification of AO type C sacral fractures.

AO spine classification	Open (*n* = 21)	Percutaneous (*n* = 27)	*p* value
C3	14	18	
Non-C3			
C0	6	7	
C1	1	2	
Total	21	27	1.00

**Table 3:** Distribution of M3 and M4 modifiers in type C fractures.

Modifier	Open C3	Percutaneous C3	Open non-C3	Percutaneous non C-3	*p* value
M3	3	10	0	1	
M4	1	0	0	0	
M3 + M4	1	3	0	1	
No modifier	9	5	7	7	
Total	14	8	7	9	0.14

**Table 4:** Associated traumatic injuries.

	Open (*n* = 21)	Percutaneous (*n* = 27)	Total (*n* = 48)	*p* value
Traumatic brain injury	2/21 (10%)	3/27 (11%)	5/48 (10%)	1.00
Closed head injury	2/21 (10%)	6/27 (22%)	8/48 (17%)	0.44
Extremity fracture	13/21 (62%)	18/27 (67%)	31/48 (65%)	0.77
Anterior pelvic ring fracture	4/21 (19%)	17/27 (63%)	21/48 (44%)	**0.003**
Acetabulum fracture	5/21 (24%)	8/27 (30%)	13/48 (27%)	0.75
Thoracic injury	9/21 (43%)	12/27 (44%)	21/48 (44%)	1.00
Blunt abdominal injury	4/21 (19%)	7/27 (26%)	11/48 (23%)	0.73
Associated spine injury	7/21 (33%)	10/27 (37%)	17/48 (35%)	1.00

*Note:* Bold values indicate statistical significance (*p* < 0.05).

**Table 5:** Postoperative outcomes.

	Open (*n* = 23)	Percutaneous (*n* = 28)	*p* value
Pelvic incidence (degrees)	68.9	65.1	0.15
Lumbar lordosis (degrees)	55.3	51.2	0.20
Surgical site infection	4	0	**0.03**
Operative time (minutes)	283.8	167.7	**< 0.01**
Estimated blood loss (mL)	678.6	81.7	**< 0.01**
Hardware removal	5	11	0.35
Postoperative transfusion	6	8	0.94
Length of stay (days)	16.9	18.2	0.62
Length of follow-up (months)	11.8	10.4	0.64
OR charges (USD)	111,310	88,573	**0.04**
OR cost (USD)	35,097	23,743	**0.01**

*Note:* Note that surgical site infections, operative time, estimated blood loss, and operative costs/charges were significantly reduced in the percutaneous cohort. Bold values indicate statistical significance (*p* < 0.05).

## Data Availability

The data that support the findings of this study are available from the corresponding author upon reasonable request.
